# The normal reference values and estimation formulae of renal structural parameters in Chinese children based on large-sample CT data

**DOI:** 10.3389/fped.2023.1174310

**Published:** 2023-07-17

**Authors:** Yong Qin, En Liu, Xiaoying Ni, Zhongxin Huang, Lu Tian, Xiaoya He, Jinhua Cai, Qiu Li

**Affiliations:** ^1^ Department of Radiology, Ministry of Education Key Laboratory of Child Development and Disorders, National Clinical Research Center for Child Health and Disorders, China International Science and Technology Cooperation Base of Child Development and Critical Disorders, Chongqing Key Laboratory of Pediatrics, Children’s Hospital of Chongqing Medical University, Chongqing, China; ^2^ Department of Gastroenterology, Second Affiliated Hospital of Army Medical University, Chongqing, China; ^3^ Department of Nephrology, Ministry of Education Key Laboratory of Child Development and Disorders, National Clinical Research Center for Child Health and Disorders, China International Science and Technology Cooperation Base of Child Development and Critical Disorders, Chongqing Key Laboratory of Pediatrics, Children’s Hospital of Chongqing Medical University, Chongqing, China

**Keywords:** kidney, structural parameter, CT, reference value, children, Chinese

## Abstract

**Purpose:**

Our aim was to investigate the normal reference value and to establish an estimation formulae for renal structural parameters (RSPs) based on large-sample CT data of Chinese children, which can provide a data reference for the clinical assessment of kidney development and diseases in Chinese children.

**Materials and Methods:**

A total of 438 children aged 0–17 years with normal renal CT images and basic indices were continuously collected. The bilateral RSP, including renal length (RL), renal width (RW), renal thickness (RT), renal volume (RV), renal cortical thickness (RCT), renal artery diameter (RAD) and renal CT value, were measured. Kendall's rank correlation was used to analyze the correlation between RSP and sex. Pearson's correlation was used to analyze the correlation between RSP and age, height and weight. Differences in the RSP of bilateral kidneys were analyzed via a paired samples t-test. Multiple linear regression was used to analyze the multivariate relationships between RSP and basic indices and establish the estimation formula of RSP.

**Results:**

The RSP of normal kidneys showed a dynamic increasing trend with age, except for the CT values. The reference value ranges (95% confidence interval) of normal RSP for each age group were determined. Pearson correlation analysis demonstrated strong correlations between RSP (RL, RW, RT, RV, RCT and, RAD) and basic indices (age, height and, weight), with height exhibiting the greatest correlation coefficient, followed by age or weight. Kendall's analysis showed that none of the RSPs were correlated with sex. The RL, RW, RV and RAD of the left kidney were larger than those of the right kidney, and the RT and RCT of the right kidney exhibited opposite results. Multiple linear regression analysis demonstrated a significant linear relationship between the RSP (RL, RW, RT, RV and, RCT) and the variables of the basic indices. The estimation formulae for calculating the RSP were established.

**Conclusion:**

This is the first Chinese study to report of the trends, normal reference values and estimation formulae of normal RSP based on large-sample CT data. These results can provide data references for assessing adequate kidney growth or disease damage in Chinese children.

## Introduction

Childhood is a critical period of kidney development. Compared with adults, the renal structural parameters (RSP) of children change dynamically with age (1). Kidney diseases such as renal hypoplasia, glomerulonephritis, infections, and hematological diseases may cause morphological changes in the kidney (2–6). To accurately evaluate the morphological changes in children's kidneys in different age groups, we must refer to a normal reference range of kidney structure or an estimation formula of kidney structure parameters.

In recent years, with the development of imaging technology (CT, MRI, ultrasound and nuclear medicine), these methods, have been applied to display the renal structure and to assess the normal development or disease state (7–12). By using these imaging methods, some researchers have measured the morphology of kidneys in children and established the criteria for normal kidney morphology, especially concerning normal size range. However, the criteria in these reports are not necessarily universal because of the influence of ethnic factors on the morphological characteristics of the kidney (13–17).

To the best of our knowledge, although China has nearly 1/6 of the world's child population under 18-years-old, there is still a lack of complete normal reference data on the RSP of the kidney. Shi et al. (13). reported an article on imaging measurements of kidney morphology by using ultrasound in a Chinese population; however, the study assessed only one measure of kidney volume (RV) and was limited to ages under 12 years. Moreover, considering that CT is currently still one of the most important renal imaging methods in children (10, 18), it is necessary to use CT imaging data to measure the developing kidneys of children to establish the normal value reference index of renal structures in Chinese children.

Therefore, the current present study aimed to examine the trends of normal RSP with age and to investigate the reference value range of each structural parameter for each age and ultimately establish the estimation formulae for calculating the RSP of the standard kidney by measuring a large sample of CT data in Chinese children of different ages. This study can provide a data reference for the clinical assessment of kidney development and diseases in Chinese children.

## Methods

### Ethical safety

This study complied with the ethical and moral requirements of medical research in the Declaration of Helsinki. The exemption of informed consent for the use of children's age, gender, and contrast CT images was approved by the Ethics Committee of Children's Hospital Affiliated to Chongqing Medical University (China), and the ethics approval number is 2022384.

### Data acquisition

The enhanced CT images were obtained on a 64-slice spiral CT (Highspeed VCT, GE, USA) or a Philips 256-slice iCT (Brilliance iCT, Holland). GE Centricity PACS was used to collect and retrieve the CT images. The hospital's medical record management system was used to collect the subject's demographic index, anthropometric indices, medical history, blood and urine renal function, regional information and parental ethnicity. Furthermore, the Philips Extended Brilliance Workspace 4.5 workstation was used to measure the RSP.

### Inclusion and exclusion criteria for the study subjects

Inclusion criteria: (1) The subjects underwent abdominal CT contrast-enhanced examinations at the Children's Hospital of Chongqing Medical University (China) between January 2012 and September 2022. (2) The patient's age was between 0 and 18 years old. (3) The demographic indices (age and gender) and anthropometric indices (height and weight) of the subjects were complete. (4) The renal serum and urine biochemical indicators of the subjects were both normal. (5) The enhanced CT images of the bilateral kidneys were complete, the thickness of the original images used to reconstruct and measure the RSP was less than 1.25 mm, and the images of the kidneys were clear.

Exclusion criteria: (1) Subjects who had a history of prematurity, malnutrition, long-term chronic wasting disease, congenital diseases of other organ systems, renal disease, renal surgery, or other diseases affecting renal structure and function were excluded. (2) The study subjects were mixed-race children. (3) The contrast CT images showed renal abnormalities in morphology or intensity, such as dysplasia, renal cysts, nephritis and tumors, etc. (4) The kidney images had significant artifacts that affected the measurement results.

### Measurement of RSP

In the Philips Extended Brilliance Workspace 4.5 workstation, the contrast CT images of the 438 subjects were imported, and the RSP was measured. These measurement procedures were performed by three imaging physicians with more than 5 years of imaging experience, whereas three additional imaging specialists with more than 10 years of imaging experience participated in the measurement process as observers. Finally, the average of the three measurements was used as the final result of the parameters for the statistical analysis.

The measurement of each parameter is explained below. Based on the CT plain scan images, the CT value was measured in the region of interest in the renal parenchymal zone, and the average value of the three regions was measured as the final renal CT value. Based on the enhanced arterial phase images of the kidney, CT multiplanar reconstruction (MPR) was used to reconstruct the cross-sectional image of the middle segment of the renal artery, and its anterior and posterior diameters were measured as RAD. Based on the enhanced cortical phase image of the kidney, MPR was used to reconstruct the short-axis image through the renal hilum; in addition, RCT at 3 points was measured in the outer cortex area; and the average value was taken as the RCT. On the reconstructed axial images at the level of the hilum, the distance between the anterior edge and the posterior edge of the kidney was defined as the RT, and the distance between the inner edge and the outer edge of the kidney was measured as the renal width. Based on the enhanced cortical phase images, the MPR was used to reconstruct the coronal slices, and the distance between the highest point and the lowest point of the kidney at the middle slice was measured as RL. Based on renal parenchymal phase images, we used the volume measurement software installed in the postprocessing workstation to measure RV.

### Statistical analysis

R software (version 3.4, http://www.Rproject.org) was used for the statistical analysis. The mean value and its 95% CI were used to analyze the trends and reference value range of each structural parameter of the kidneys with age. The Kendall's rank correlation was used to analyze the correlation between RSP and sex. The Pearson's correlation was used to analyze the correlation between RSP and age, height and weight. Moreover, multiple linear regression was used to analyze the multivariate relationships between the RSP (RL, RW, RT, RV, RCT and, RAD) and basic indices (sex, age, height and weight) and to establish the estimation formula for calculating the RSP of the standard kidney. Scatter plots were used to test the fit of the regression equation, with the predicted values of the equation as the horizontal coordinates and the actual values as the vertical coordinates. Differences in the RSP between the right and left kidneys were comparatively analyzed by using a paired-sample t test. All of the tests were considered to be statistically significant at the significance level of *P* < 0.05 and to be highly significant at the significance level of *P* < 0.01.

## Results

### Population data

Based on the inclusion and exclusion criteria, 107 children who did not meet the requirements were excluded and 438 subjects aged 0–17 years with normal renal contrast CT images were included in the study. The demographic and basic indices (sex, age, height and weight) of the 438 included subjects are shown in Supplementary Table S1.

The RSPs of the bilateral kidneys were accurately measured and recorded. The necessary reasons for abdominal CT examinations included acute abdominal trauma (46.8%, 205/438), acute abdominal pain pending investigation (24.4%, 107/438), acute abdominal infection (mainly appendicitis) (21.3%, 93/438) and other rare reasons (7.5%, 33/438), such as acute intestinal obstruction and foreign bodies in the digestive tract. To assess the trends in renal structure parameters with age, the 438 children were divided into 17 groups (with one group spanning 1 year of age).

### Trends and reference range

In general, the RSP (RL, RW, RT, RV, RCT and RAD) exhibited a dynamic increasing trend with age (Supplementary Image S1.1–S1.6). Specifically, RT and RAD showed a steadily increasing rate from birth to ∼17 years (Supplementary Image S1.3, S1.6). Additionally, RL, RW, RV and RCT showed rapid growth rates from birth to ∼13 years; however, their growth rates slowed down significantly after ∼13 years (Supplementary Image S1.1, S1.2, S1.4, S1.5). Furthermore, the CT value showed no significant trend with age (Supplementary Image S1.7), and its approximate reference value range (95% CI) was between 34∼43 HU.

The reference ranges (95% CI) of each renal structural parameter (RL, RW, RT, RV, RCT and RAD) for all of the age groups are displayed in Tables 1–6.

**Table 1:** Trends and reference range of renal length with age.

Age (year)	*n*	Renal length (mm, $\bar{x}\pm s$)		95% CI	
		L-kidney	R-kidney	L-kidney	R-kidney
>0, ≤1	30	55.61 ± 6.99	54.61 ± 5.49	53.11∼58.11	52.65∼56.57
>1, ≤2	29	63.79 ± 6.49	63.40 ± 5.04	61.43∼66.15	61.57∼65.24
>2, ≤3	25	68.50 ± 8.24	68.64 ± 4.19	65.27∼71.73	67.00∼70.28
>3, ≤4	41	69.29 ± 6.66	69.56 ± 5.67	67.25∼71.33	67.83∼71.30
>4, ≤5	23	77.06 ± 11.51	74.83 ± 7.75	72.36∼81.76	71.66∼78.00
>5, ≤6	40	79.80 ± 6.52	78.10 ± 5.96	77.77∼81.82	76.25∼79.95
>6, ≤7	25	81.98 ± 8.78	82.60 ± 7.84	78.54∼85.42	79.53∼85.68
>7, ≤8	25	85.02 ± 7.13	81.35 ± 6.35	82.23∼87.81	78.86∼83.84
>8, ≤9	30	85.35 ± 9.53	85.19 ± 7.97	81.94∼88.76	82.34∼88.04
>9, ≤10	24	89.27 ± 9.40	86.85 ± 7.25	85.51∼93.03	83.95∼89.75
>10, ≤11	30	92.14 ± 10.26	89.55 ± 8.41	88.47∼95.81	86.54∼92.56
>11, ≤12	44	94.21 ± 10.85	92.95 ± 9.94	91.01∼97.42	90.01∼95.89
>12, ≤13	28	99.05 ± 10.36	98.24 ± 8.83	95.21∼102.88	94.97∼101.51
>13, ≤14	21	100.86 ± 11.94	99.08 ± 9.09	95.76∼105.97	95.19∼102.96
>14, ≤15	13	101.18 ± 15.45	100.49 ± 13.09	92.78∼109.58	93.38∼107.61
>15, ≤16	5	101.98 ± 6.92	101.84 ± 9.06	95.92∼108.04	93.90∼109.78
>16, ≤17	5	102.12 ± 8.13	101.72 ± 8.84	94.99∼109.25	93.98∼109.46

**Table 2:** Trends and reference range of renal width with age.

Age (year)	*n*	Renal width (mm, $\bar{x}\pm s$)		95% CI	
		L-kidney	R-kidney	L-kidney	R-kidney
>0, ≤1	30	27.12 ± 3.84	26.12 ± 3.14	25.75∼28.50	25.00∼27.25
>1, ≤2	29	30.33 ± 2.83	28.27 ± 2.52	29.30∼31.36	27.35∼29.18
>2, ≤3	25	30.86 ± 2.97	29.10 ± 2.81	29.69∼32.02	28.00∼30.20
>3, ≤4	41	31.79 ± 4.62	30.40 ± 3.86	30.38∼33.21	29.22∼31.59
>4, ≤5	23	33.81 ± 6.09	32.00 ± 4.25	31.32∼36.30	30.26∼33.73
>5, ≤6	40	35.74 ± 4.23	33.82 ± 2.81	34.43∼37.06	32.95∼34.70
>6, ≤7	25	37.33 ± 3.80	35.18 ± 3.99	35.84∼38.82	33.61∼36.74
>7, ≤8	25	38.20 ± 5.43	35.74 ± 3.09	36.07∼40.33	34.52∼36.95
>8, ≤9	30	38.65 ± 4.83	36.45 ± 3.27	36.92∼40.38	35.28∼37.62
>9, ≤10	24	39.08 ± 4.29	37.66 ± 3.81	37.37∼40.80	36.14∼39.19
>10, ≤11	30	40.96 ± 5.19	39.45 ± 3.83	39.10∼42.82	38.08∼40.82
>11, ≤12	44	42.21 ± 4.95	39.86 ± 4.60	40.75∼43.68	38.50∼41.22
>12, ≤13	28	43.48 ± 4.39	42.06 ± 5.79	41.85∼45.10	39.92∼44.20
>13, ≤14	21	43.53 ± 3.09	42.59 ± 3.71	42.21∼44.85	41.00∼44.17
>14, ≤15	13	43.68 ± 4.11	42.70 ± 3.82	41.44∼45.91	40.62∼44.78
>15, ≤16	5	43.84 ± 2.69	43.18 ± 2.29	41.48∼46.20	41.17∼45.19
>16, ≤17	5	44.00 ± 2.05	43.82 ± 3.48	42.20∼45.80	40.77∼46.87

**Table 3:** Trends and reference range of renal thickness with age.

Age (year)	*n*	Renal thickness (mm, $\bar{x}\pm s$)		95% CI	
		L-kidney	R-kidney	L-kidney	R-kidney
>0, ≤1	30	32.00 ± 4.22	32.18 ± 4.33	30.49∼33.51	30.63∼33.73
>1, ≤2	29	37.42 ± 2.41	37.60 ± 2.75	36.54∼38.30	36.60∼38.61
>2, ≤3	25	38.73 ± 3.90	39.58 ± 3.08	37.20∼40.26	38.38∼40.79
>3, ≤4	41	39.37 ± 2.96	40.76 ± 3.76	38.46∼40.28	39.61∼41.91
>4, ≤5	23	42.27 ± 4.50	42.33 ± 5.51	40.43∼44.10	40.07∼44.58
>5, ≤6	40	42.52 ± 4.06	42.81 ± 4.56	41.26∼43.78	41.40∼44.23
>6, ≤7	25	44.07 ± 2.61	44.00 ± 3.96	43.05∼45.10	42.44∼45.55
>7, ≤8	25	44.14 ± 5.04	44.80 ± 3.60	42.17∼46.11	43.39∼46.22
>8, ≤9	30	45.82 ± 4.03	45.93 ± 3.13	44.38∼47.27	44.81∼47.05
>9, ≤10	24	46.35 ± 5.65	46.95 ± 4.73	44.09∼48.62	45.06∼48.84
>10, ≤11	30	48.04 ± 3.83	48.79 ± 6.53	46.67∼49.41	46.45∼51.12
>11, ≤12	44	50.31 ± 5.00	50.55 ± 4.59	48.83∼51.79	49.19∼51.90
>12, ≤13	28	51.59 ± 5.54	51.92 ± 5.93	49.53∼53.64	49.72∼54.12
>13, ≤14	21	52.63 ± 3.05	53.65 ± 4.00	51.32∼53.93	51.94∼55.36
>14, ≤15	13	53.61 ± 6.80	54.21 ± 7.13	49.91∼57.30	50.33∼58.08
>15, ≤16	5	54.20 ± 4.00	55.04 ± 4.18	50.69∼57.71	51.37∼58.71
>16, ≤17	5	54.52 ± 3.05	55.32 ± 4.38	51.84∼57.20	51.48∼59.16

**Table 4:** Trends and reference range of renal volume with age.

Age (year)	n	Renal volume (cm^3^, $\bar{x}\pm s$)		95% CI	
		L-kidney	R-kidney	L-kidney	R-kidney
>0, ≤1	30	30.76 ± 8.73	29.51 ± 7.16	27.03∼34.50	26.45∼32.58
>1, ≤2	29	43.76 ± 7.79	41.62 ± 8.61	40.06∼47.46	37.53∼45.71
>2, ≤3	25	46.69 ± 8.72	44.52 ± 7.69	42.42∼50.97	40.75∼48.28
>3, ≤4	41	50.89 ± 9.64	49.79 ± 8.99	47.18∼54.59	46.33∼53.24
>4, ≤5	23	61.87 ± 12.62	54.79 ± 10.96	54.73∼69.01	48.59∼60.99
>5, ≤6	40	68.81 ± 14.05	64.86 ± 13.78	63.41∼74.21	59.57∼70.16
>6, ≤7	25	80.79 ± 15.45	78.54 ± 12.73	72.70∼88.89	71.87∼85.21
>7, ≤8	25	80.12 ± 25.12	74.39 ± 19.82	68.82∼91.42	65.48∼83.31
>8, ≤9	30	86.16 ± 16.62	80.47 ± 14.28	80.00∼92.31	75.18∼85.76
>9, ≤10	24	97.36 ± 17.24	91.97 ± 14.47	89.39∼105.32	85.29∼98.66
>10, ≤11	30	101.98 ± 21.12	94.69 ± 23.31	92.72∼111.24	84.47∼104.90
>11, ≤12	44	114.42 ± 26.86	106.76 ± 23.91	104.97∼123.88	98.34∼115.18
>12, ≤13	28	129.09 ± 23.54	124.91 ± 24.23	117.17∼141.00	112.65∼137.17
>13, ≤14	21	128.86 ± 13.65	124.13 ± 15.72	122.56∼135.17	116.86∼131.39
>14, ≤15	13	139.00 ± 38.05	136.24 ± 30.89	114.14∼163.86	116.06∼156.43
>15, ≤16	5	137.51 ± 17.47	135.86 ± 19.02	122.20∼152.82	119.18∼152.53
>16, ≤17	5	138.34 ± 17.72	137.43 ± 15.57	125.18∼147.49	123.78∼151.08

**Table 5:** Trends and reference range of renal cortical thickness with age.

Age (year)	*n*	Renal cortical thickness (mm, $\bar{x}\pm s$)		95% CI	
		L-kidney	R-kidney	L-kidney	R-kidney
>0, ≤1	30	3.08 ± 0.79	3.13 ± 1.03	2.80∼3.36	2.76∼3.49
>1, ≤2	29	3.72 ± 0.62	3.75 ± 0.64	3.49∼3.94	3.52∼3.99
>2, ≤3	25	4.00 ± 0.66	4.07 ± 0.68	3.74∼4.26	3.80∼4.33
>3, ≤4	41	4.25 ± 0.83	4.47 ± 0.81	4.00∼4.51	4.22∼4.72
>4, ≤5	23	4.54 ± 0.88	4.57 ± 0.81	4.18∼4.90	4.24∼4.90
>5, ≤6	40	4.96 ± 0.92	5.00 ± 0.73	4.67∼5.24	4.78∼5.23
>6, ≤7	25	5.03 ± 1.16	5.30 ± 1.00	4.58∼5.49	4.91∼5.69
>7, ≤8	25	5.39 ± 0.86	5.37 ± 0.77	5.05∼5.73	5.06∼5.67
>8, ≤9	30	5.42 ± 0.96	5.53 ± 1.11	5.08∼5.76	5.13∼5.92
>9, ≤10	24	5.50 ± 1.14	5.63 ± 0.69	5.05∼5.96	5.36∼5.91
>10, ≤11	30	5.77 ± 1.23	5.74 ± 1.29	5.33∼6.21	5.28∼6.20
>11, ≤12	44	5.87 ± 1.13	6.12 ± 1.21	5.53∼6.20	5.76∼6.48
>12, ≤13	28	5.92 ± 1.18	6.23 ± 1.08	5.48∼6.36	5.83∼6.63
>13, ≤14	21	6.36 ± 0.83	6.53 ± 0.98	6.00∼6.72	6.11∼6.95
>14, ≤15	13	6.42 ± 0.98	6.62 ± 1.23	5.88∼6.95	5.95∼7.29
>15, ≤16	5	6.42 ± 0.80	6.64 ± 0.85	5.71∼7.13	5.90∼7.38
>16, ≤17	5	6.52 ± 0.68	6.67 ± 0.46	5.93∼7.11	6.27∼7.07

**Table 6:** Trends and reference range of renal artery diameter with age.

Age (year)	*n*	Renal artery diameter (mm, $\bar{x}\pm s$)		95% CI	
		L-kidney	R-kidney	L-kidney	R-kidney
>0, ≤1	30	2.52 ± 0.76	2.65 ± 0.86	2.25∼2.80	2.34∼2.96
>1, ≤2	29	2.98 ± 0.93	2.98 ± 0.85	2.64∼3.32	2.68∼3.29
>2, ≤3	25	3.25 ± 1.02	3.06 ± 0.94	2.85∼3.65	2.69∼3.42
>3, ≤4	41	3.27 ± 1.03	3.15 ± 0.93	2.96∼3.59	2.87∼3.43
>4, ≤5	23	3.41 ± 0.96	3.26 ± 0.92	3.02∼3.80	2.89∼3.64
>5, ≤6	40	3.72 ± 1.64	3.46 ± 1.10	3.22∼4.23	3.12∼3.80
>6, ≤7	25	3.85 ± 1.11	3.58 ± 1.04	3.41∼4.28	3.18∼3.99
>7, ≤8	25	3.86 ± 1.50	3.86 ± 1.29	3.27∼4.44	3.36∼4.36
>8, ≤9	30	4.13 ± 1.55	3.91 ± 1.62	3.58∼4.69	3.33∼4.49
>9, ≤10	24	4.40 ± 1.12	4.13 ± 0.99	3.95∼4.84	3.73∼4.53
>10, ≤11	30	4.44 ± 1.76	4.21 ± 1.33	3.81∼5.07	3.73∼4.69
>11, ≤12	44	4.63 ± 1.30	4.36 ± 1.39	4.24∼5.01	3.95∼4.77
>12, ≤13	28	4.71 ± 1.59	4.55 ± 1.56	4.13∼5.30	3.97∼5.13
>13, ≤14	21	4.75 ± 1.01	4.72 ± 1.48	4.32∼5.19	4.09∼5.36
>14, ≤15	13	5.02 ± 1.22	4.84 ± 0.75	4.35∼5.68	4.43∼5.24
>15, ≤16	5	5.08 ± 0.68	4.94 ± 0.87	4.48∼5.68	4.18∼5.70
>16, ≤17	5	5.12 ± 0.91	4.96 ± 0.93	4.32∼5.92	4.15∼5.77

### Correlation analysis

The Kendall's analysis showed that none of the bilateral RSPs were correlated with sex.

**Figure 1:**
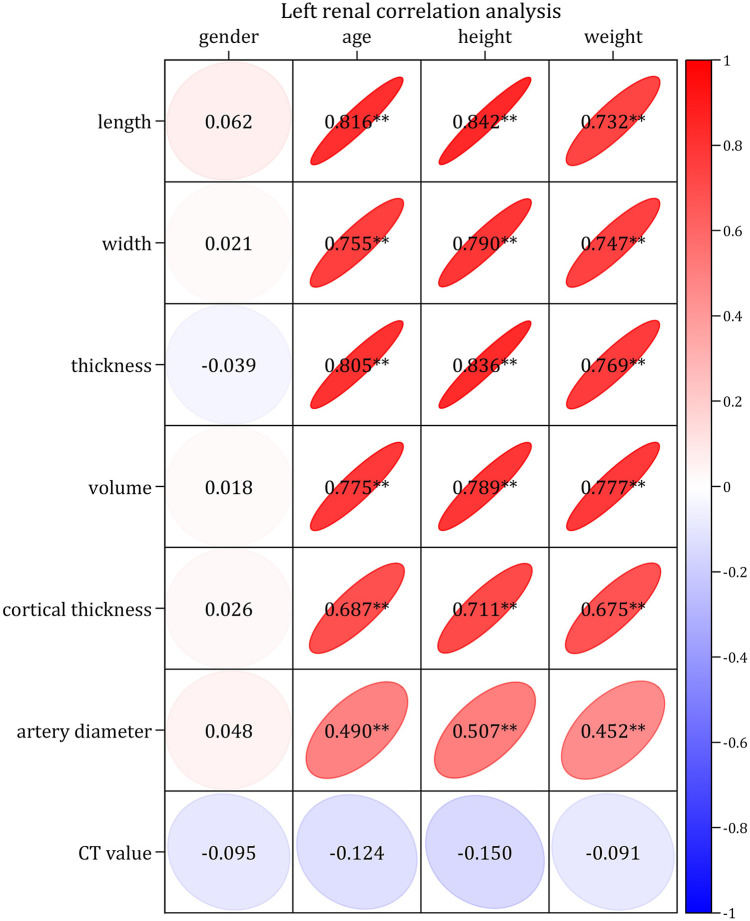
Correlation diagram of left renal parameters with basic indices.

**Figure 2:**
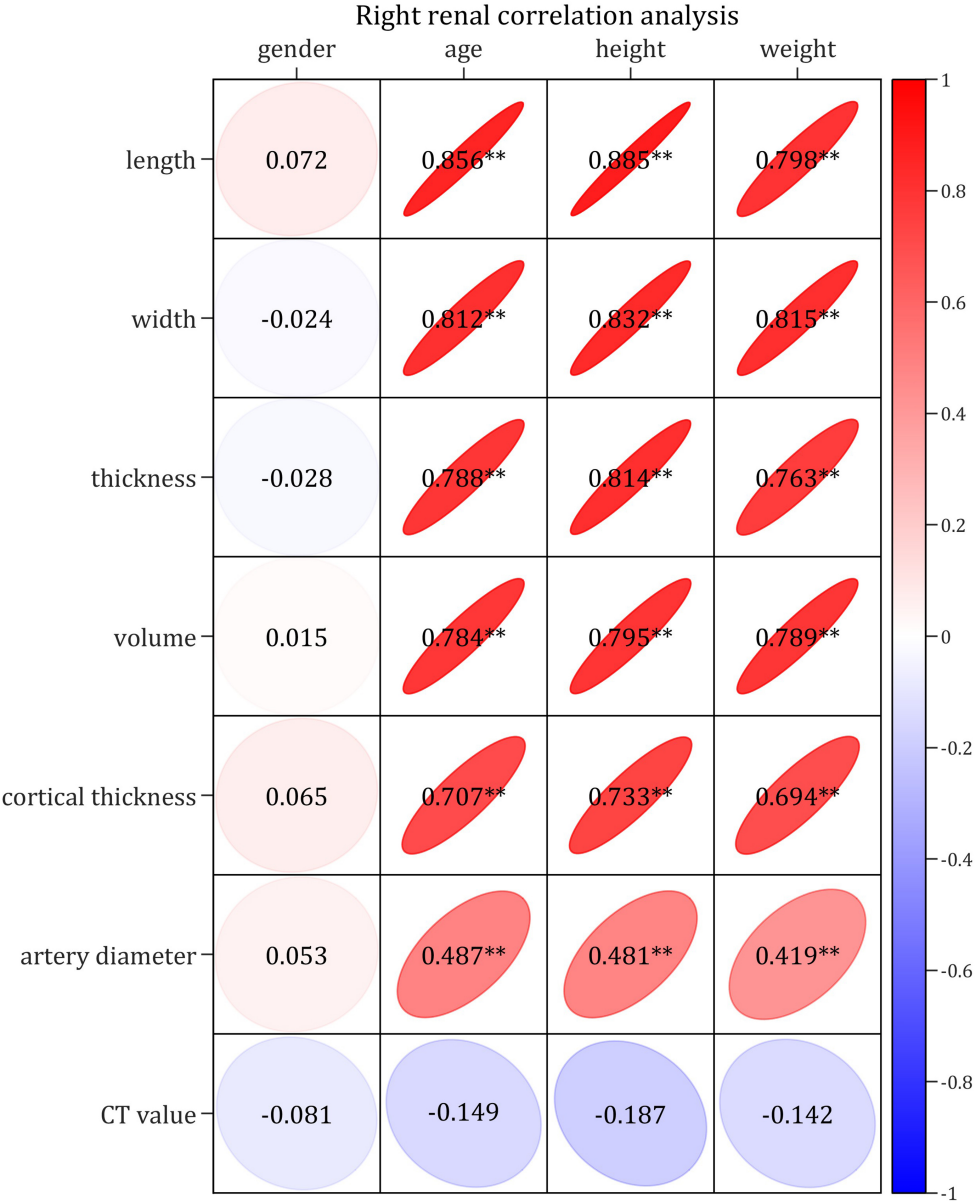
Correlation diagram of right renal parameters with basic indices.

**Figure 3:**
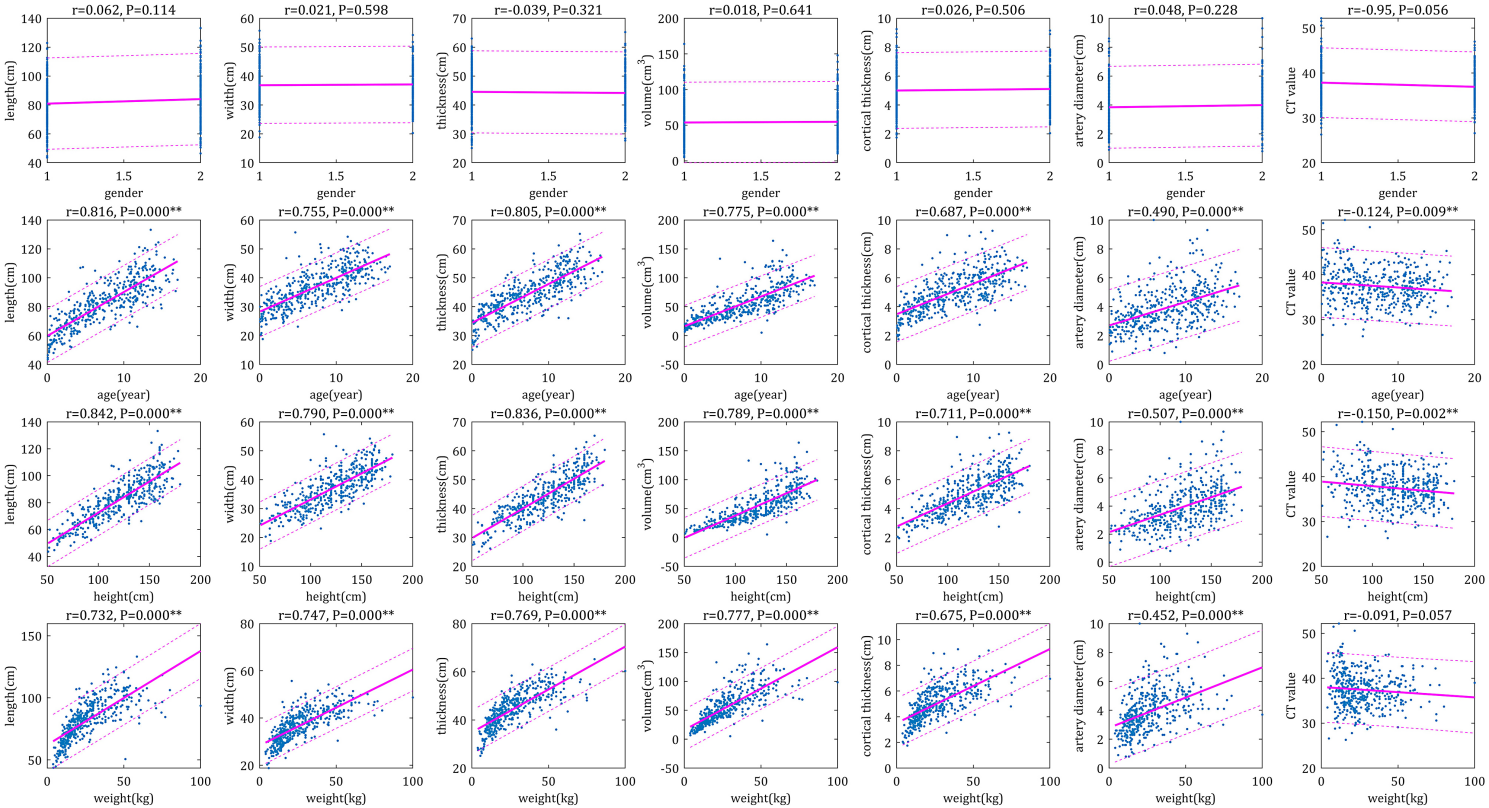
Scatter plots of correlation left renal parameters with basic indices. The physiological parameters were used as horizontal coordinates and the individual parameters of the right renal were used as vertical coordinates to make correlation plots, the closer the graph was to the regression line, the stronger the correlation was.

**Figure 4:**
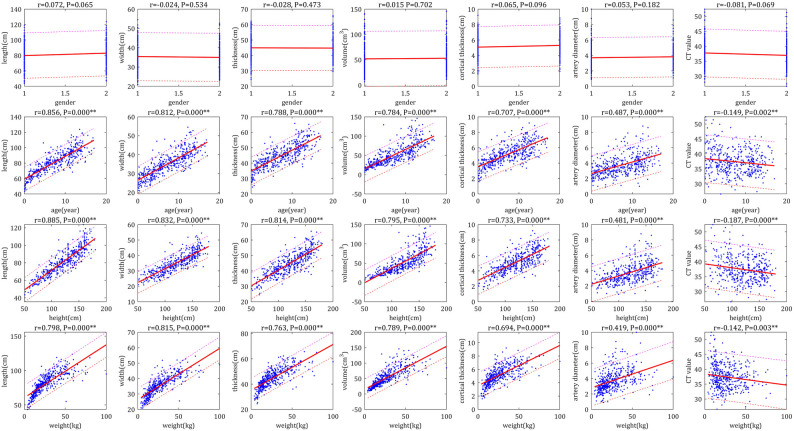
Scatter plots of correlation of right renal parameters with basic indices. The physiological parameters were used as horizontal coordinates and the individual parameters of the left renal were used as vertical coordinates to make correlation plots, the closer the graph was to the regression line, the stronger the correlation was.

The Pearson correlation analysis demonstrated very strong (correlation coefficients > 0.8) or strong correlations (correlation coefficients > 0.6) between the bilateral RSP (RL, RW, RT, RV and RCT) and age, height, and weight, respectively; moreover, there were moderately strong correlations (correlation coefficient > 0.4) between RAD and age, height and weight, respectively, as well as no correlation (correlation coefficients < 0.2) between CT values and age, height and weight, respectively (Figures 1–4). In addition, based on the level of the correlation coefficient, the statistics also showed that height had the greatest correlation with the RSP (RL, RW, RT, RV and RCT), followed by age and weight (Figures 1–4).

### Multiple linear regression analysis and estimation formulae

The multiple linear regression analysis demonstrated that there was a significant linear relationship between the bilateral RSP (RL, RW, RT, RV, RCT and RAD) and the variables of the basic indices (gender, age, height and weight) (Supplementary Tables S2, S3).

**Figure 5:**
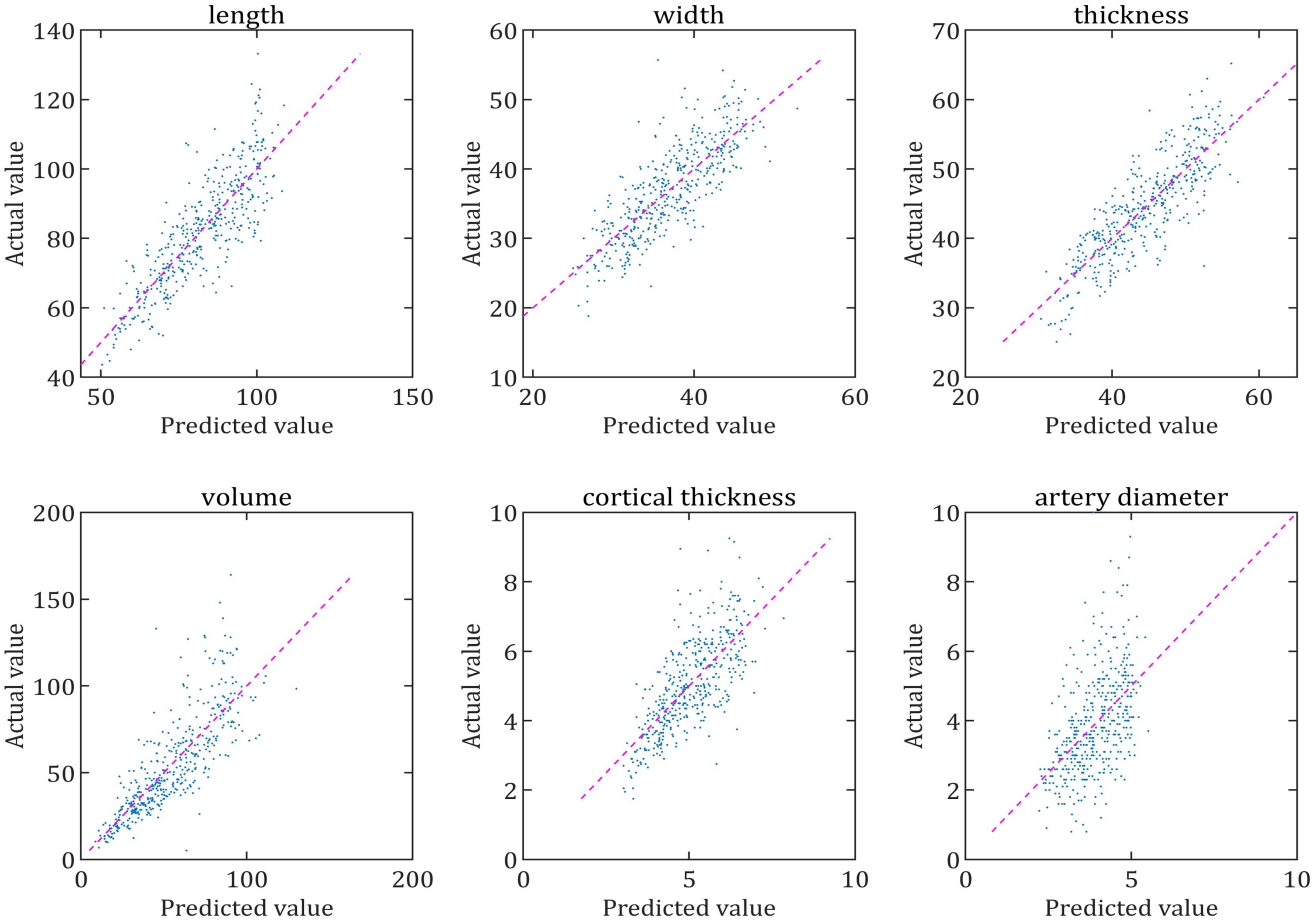
Comparison of the predicted and actual values of each parameter of the left renal. The scatter plot is made with the predicted value of the regression equation as the horizontal coordinate and the actual value as the vertical coordinate. The closer the scatter plot is to the diagonal line, the better the equation is fitted and the closer the predicted value of the equation is to the actual value.

**Figure 6:**
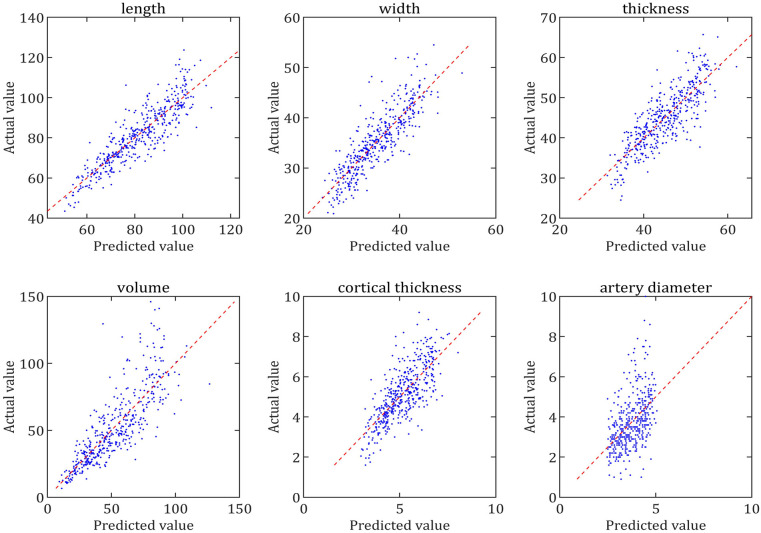
Comparison of the predicted and actual values of each parameter of the right renal. The scatter plot is made with the predicted value of the regression equation as the horizontal coordinate and the actual value as the vertical coordinate. The closer the scatter plot is to the diagonal line, the better the equation is fitted and the closer the predicted value of the equation is to the actual value.

The scatter plots, which aime to test the fit of the regression equation, showed that the scatter plots for RL, RW, RT, RV and RCT were very close to the diagonal, which indicated that the predicted values of the equations were very close to the actual values and ultimately indicated that the equations had a very good fit, whereas the fit for RAD was relatively poor (Figures 5, 6).

The estimation formulae for calculating the RSP of standard kidneys in the children were initially established, as shown in Table 7. Overall, it can be used to provide a data reference for the clinical evaluation of children's renal development and disorders.

**Table 7:** Multiple linear regression equation for each structural parameter.

Structural parameters	Left kidney		Right kidney	
	regression equation	P	regression equation	P
Renal length (mm)	=1.275*gender + 0.409*age + 0.404*height −0.002*weight + 28.507	**	=1.3*gender + 0.283*age + 0.36*height +0.112*weight + 30.619	**
Renal width (mm)	=−0.433*gender−0.057*age + 0.14*height +0.107*weight + 18.173	**	=−1.187*gender + 0.215*age + 0.081*height +0.15*weight + 21.46	**
Renal thickness (mm)	=−1.214*gender + 0.072*age + 0.16*Height +0.08*weight + 24.063	**	=−1.011*gender + 0.151*age + 0.137*height +0.105*weight + 25.817	**
Renal volume (cm^3^)	=−0.402*gender + 1.235*age + 0.521*height +0.903*weight−14.043	**	=−1.207*gender + 1.229*age + 0.464*height +0.875*weight−9.897	**
Renal cortical thickness (mm)	=−0.022*gender + 0.016*age + 0.021*height −0.02*weight + 1.905	**	=0.084*gender + 0.01*age + 0.023*height +0.02*weight + 1.639	**
Renal artery diameter (mm)	=0.051*gender + 0.015*age + 0.021*height +0.004*weight + 1.094	**	=0.029*gender + 0.099*age + 0.008*height −0.001*weight + 2.027	**

Gender: mal = 1, femal = 2; age: year; height: cm; weight: kg.

**P < 0.01, indicating that the regression equation is highly significant.

### Comparative analysis of the left and right kidneys

**Figure 7:**
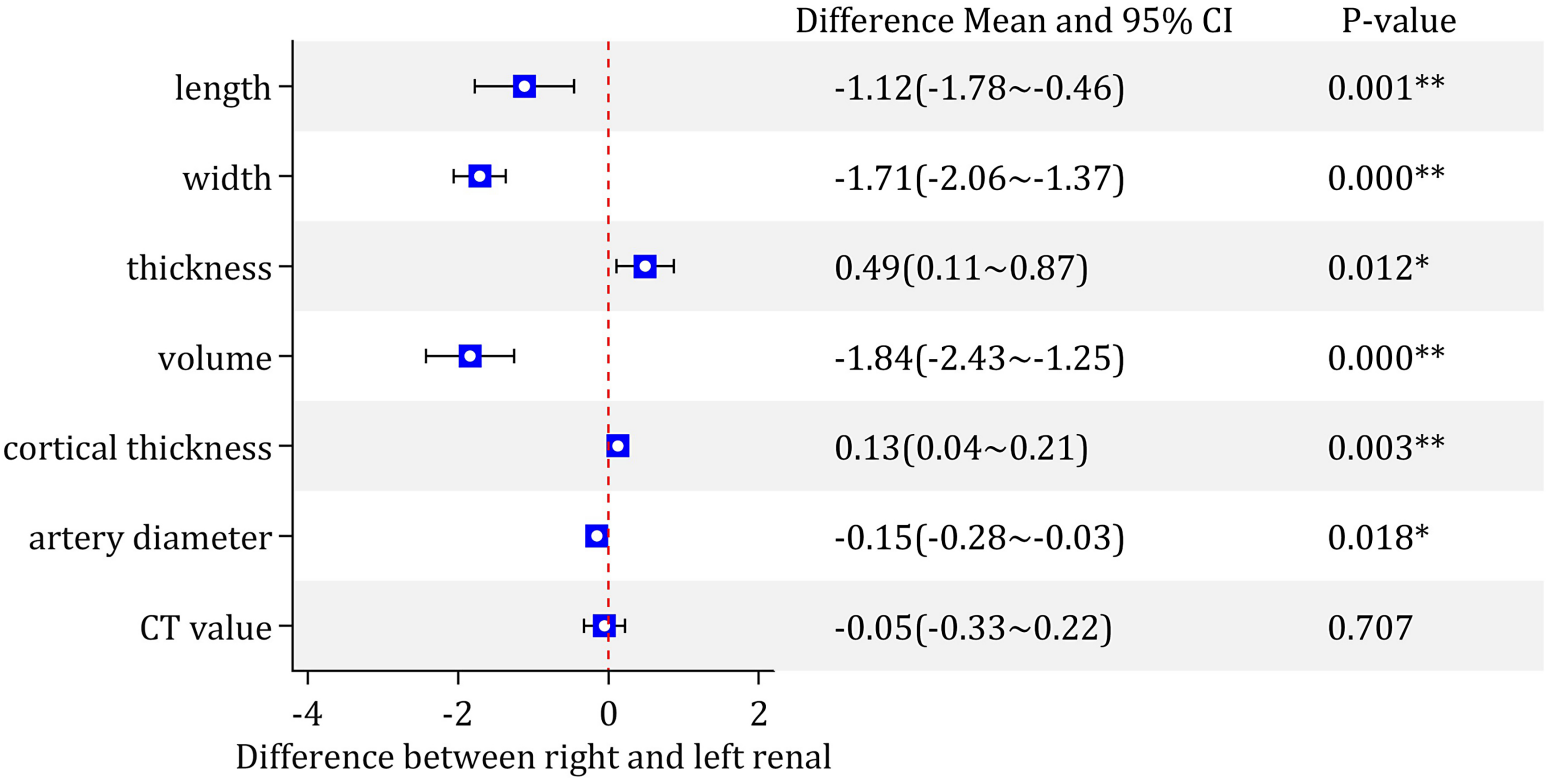
Comparison results of various parameters of the left and right kidney. *P < 0.05, significant difference between right and left renal parameters; **P < 0.01, highly significant difference between right and left renal parameters.

The RL, RW and RV of the left kidney were larger than those of the right kidney (with a highly significant difference), and the RAD of the left kidney was larger than that of the right kidney (with a significant difference) (Figure 7).

The RCT and RT of the right kidney were significantly larger than those of the left kidney (Figure 7).

There was no statistically significant difference in the CT value between the bilateral kidneys (Figure 7).

## Discussion

In children, the knowledge of normal reference values is fundamental to accurately assess kidney growth and possible disease damage. China has nearly 1/6 of the world's child population under 18-years-old; however, complete normal reference values are still lacking (13). In this study, we used a large sample of CT data to initially report on the trends and the normal reference values of each structural parameter for each age and the estimation formulae used to calculate the RSP of standard kidneys. These results can provide a data reference for assessing adequate renal growth or disease injury.

Although the majority of previous reports studying kidney size have used ultrasound, some of the disadvantages of ultrasound are that it may lead to measurement errors that cannot be ignored, such as the inability to adhere to standardized measurement methods, the influence of equipment performance and software on measurement results, the problem of intra- and interobserver reproducibility, and the difficulty and poor accuracy of measuring RV (10, 18). In contrast, although CT has well-known radiation risks, CT overcomes the problems of poor reproducibility of ultrasound and the difficulty of measuring RV. Therefore, the examination method that was used in this study was CT (rather than ultrasound), and theoretically, the normal reference values and estimation equations of the kidney based on the data measured by CT should have higher accuracy and confidence than ultrasound (9, 10).

It is well known that kidney size (RL, RW, RT and RV) is related to age, height and weight. Almost all studies have shown that height correlates best with kidney length (10, 13, 15, 16), and our findings support this conclusion, with height being the most relevant factor, followed by age and weight. Our results also showed that height correlated best with kidney width, thickness and cortical thickness, followed by age and weight. Moreover height, weight, age or BSA had the greatest correlation with kidney volume in previous studies (13, 16, 17, 19, 20), and our data support the conclusion that height has the best correlation with kidney volume (16). However, it should be noted that our study did not include BSA as a variable, which was mainly due to the controversial nature of the BSA calculation formula itself and the complexity of BSA calculations (10). As shown by our results, the absence of a correlation between sex and kidney size has also been confirmed by most reports, although a few reports have determined the opposite viewpoint (12, 21). These differences may be due to the low statistical power of a small number of study groups or differences in ethnicity.

According to the maximum correlation coefficient of the univariate analysis, previous studies have developed a number of univariate linear regression equations to estimate kidney size, such as kidney length and volume (13, 16, 17). Although univariate equations are easy to calculate, their accuracy is relatively poor. Given the accuracy of the calculations and the relatively small number of variables, this study established multiple linear regression equations to calculate all RSPs of the kidney. These equations have been statistically shown statistically to be strongly correlated between the total dependent variables and independent variables. Therefore, we recommend the use of multiple linear regression equations in practice to estimate the RSP to ensure the accuracy of the calculations.

Despite the high accuracy of the reference ranges and the estimation formulae provided in this study, there were three points that still deserve to be highlighted when these are clinically applied clinically to assess individual kidney size. First, the reference range criteria in this study cannot absolutely confirm normal or abnormal kidney size because the actual normal range of kidney size is relatively wide, and the reference range was defined by a 95% confidence interval for normal children. Therefore, in clinical practice, CT measurement of renal size using the reference ranges or estimation formulae needs to be combined with other clinical findings and/or a follow-up examination. Second, the normal range of kidney size is a dynamic concept and should be assessed separately for each individual based on their individual demographic and basic indices; therefore, the calculated results based on multiple linear regression equations may be more accurate than the reference value range. Third, the use of RV to assess kidney size has a higher sensitivity and accuracy than the use of two-dimensional parameters (length, width and thickness). This is mainly due to the individual variability of kidney morphology and the fact that changes in two-dimensional RSP caused by kidney disease are not as sensitive as changes in RV (13, 14). However, RV measurement is not always available in some hospitals. Under such conditions, it is still important to use two-dimensional parameters to determine whether the kidney size is abnormal based on the reference range of normal values.

In addition to the parameters of volume, length, width and thickness that have been the focus of most scholars, we have incidentally studied the pattern of age-related changes in RCT, RAD and kidney CT values. Although the measurement of RCTs can be used to assess renal injury (9), few articles have reported on normal reference values for children. This study provided a range of reference values for RCTs in normal children, which will provide a more meaningful clinical reference. The RAD grows thicker with age and correlates moderately with height, age, and weight. The left renal artery is thicker than the right, which may be related to the larger size of the left kidney. The normal range of RAD values can be used to assess the degree of renal artery stenosis due to various childhood vascular diseases (22, 23). Moreover, kidney CT values are not related to basic indices. The data from this study show that the CT values of the kidneys generally range from approximately 34–43 HU (95% CI). If the measured renal CT values are significantly different from this range, the presence of renal pathological changes, such as hemorrhage, inflammation, tumors and cystic lesions, should be considered.

Although some previous studies have shown no significant difference between the size of the left and right kidney (15), most studies have shown that the left kidney is larger than the right kidney, mainly in terms of kidney volume and length (12, 13). In our study, the left kidney was also significantly larger and longer than the right kidney; however, our study also demonstrated a previously unreported finding that the right kidney is thicker, and that the right kidney has a thicker cortex than the left kidney. Although we have not found a reasonable explanation for this interesting finding, it suggested that if the left kidney is larger than the right kidney, it must specifically refer to the volume, length or width of the kidney, but not to the thickness and cortical thickness of the kidney.

There were still some limitations in this study. First, our study was a single-center study, and these results may not fully reflect the kidney growth patterns of children across all regions of China. However, due to the rapid population migration and reproduction associated with China's rapid economic development, this may not be a great concern. Second, we did not compare the magnitude of error between CT and ultrasound measurements of kidney size, although there is no dispute that CT is more accurate (10). Third, because of the rapid changes in organ growth during infancy (13, 16, 24), the results of this study may not fully reflect the details of changes in the kidneys of infants due to the small sample size. Thankfully, there are some studies that have been specifically performed on infants by ultrasound that can be used as a reference (24). In addition, the absence of data for the ∼18-year group and the relatively small amount of data for the ∼16 and ∼17-year groups in this study are relatively unfortunate. In all, we will continue to collect relevant data and increase the sample size in order to further improve the accuracy of the reference values and evaluation formulas in future studies.

In conclusion, this is the first Chinese study to report on the trends and the normal reference value of each structural parameter for each age, and an estimation formulae was used to calculate the RSP of standard kidneys according to large-sample CT data aged 0–17 years. These results can provide data references for assessing adequate kidney growth or disease damage in Chinese children.

## Data Availability

The original contributions presented in the study are included in the article/**Supplementary Material**, further inquiries can be directed to the corresponding author/s.
