# Supplementary material for: The normal reference values and estimation formulae of renal structural parameters in Chinese children based on large-sample CT data
Source: Front Pediatr. 2023 Jul 17;11:1174310. doi: 10.3389/fped.2023.1174310 (PMC10388191; doi:10.3389/fped.2023.1174310)
Supplement: Supplementary file 1 [file Table1.pdf]

Supplementary Material

The normal reference values and estimation formulae of renal structural parameters in Chinese children based on large-sample CT data

Yong Qin, En Liu, Xiaoying Ni, Zhongxin Huang, Lu Tian, Xiaoya He, Jinhua Cai*, Qiu Li*.

[1]* Correspondence: Liqiu809@hospital.cqmu.edu.cn

[2]* Correspondence: cai_jinhua@126.com

Supplementary Tables

Supplementary Table 1. Demographic population data

Index	Median(min-max) or N(%)
Age	7.33(0.08-17.00)
Gender	
boy	247(56.39%)
girl	191(43.61%)
height	120.95(51.00-180.00)
weight	22.50(3.90-100.00)

Supplementary Table 2. Multiple linear regression analysis of each left renal structural parameter and basic indices

Renal length	β(SE)	95% CI	P-value
Intercept	28.507(4.160)	20.332~36.683	0.000**
gender	1.275(0.843)	-0.381~2.932	0.131
age(year)	0.409(0.338)	-0.255~1.074	0.227
height(cm)	0.404(0.054)	0.297~0.512	0.000**
weight(kg)	-0.002(0.054)	-0.109~0.104	0.967
The overall regression F=266.016, P=0.000, the regression equation is significant.			
**Renal width**	**β(SE)**	**95% CI**	**P-value**
Intercept	18.173(1.935)	14.370~21.977	0.000**
gender	-0.433(0.392)	-1.204~0.337	0.269
age(year)	-0.057(0.157)	-0.366~0.252	0.718
height(cm)	0.140(0.025)	0.090~0.190	0.000**
weight(kg)	0.107(0.025)	0.057~0.157	0.000**
The overall regression F=192.098, P=0.000, the regression equation is significant.			
**Renal thickness**	**β(SE)**	**95% CI**	**P-value**
Intercept	24.063(1.855)	20.416~27.709	0.000**
gender	-1.214(0.376)	-1.953~-0.475	0.001**
age(year)	0.072(0.151)	-0.224~0.368	0.634
height(cm)	0.160(0.024)	0.112~0.208	0.000**
weight(kg)	0.080(0.024)	0.032~0.127	0.001**
The overall regression F=268.803, P=0.000, the regression equation is significant.			

Renal volume	β(SE)	95% CI	P-value
Intercept	-14.043(8.630)	-31.027~2.941	0.105
gender	-0.402(1.770)	-3.885~3081	0.821
age(year)	1.235(0.676)	-0.096~2.567	0.069
height(cm)	0.521(0.112)	0.301~0.741	0.000**
weight(kg)	0.903(0.106)	0.694~1.111	0.000**
The overall regression F=210.913, P=0.000, the regression equation is significant.			

Renal cortical thickness	β(SE)	95% CI	P-value
Intercept	1.905(0.443)	1.035~2.776	0.000**
gender	-0.022(0.090)	-0.198~0.154	0.807
age(year)	0.016(0.036)	-0.055~0.087	0.657
height(cm)	0.021(0.006)	0.009~0.032	0.000**
weight(kg)	0.020(0.006)	0.009~0.031	0.001**
The overall regression F=116.704, P=0.000, the regression equation is significant.			

Renal artery diameter	β(SE)	95% CI	P-value
Intercept	1.094(0.596)	-0.077~2.264	0.067
gender	0.051(0.121)	-0.187~0.288	0.675
age(year)	0.015(0.048)	-0.080~0.110	0.751
height(cm)	0.021(0.008)	0.005~0.036	0.008**
weight(kg)	0.004(0.008)	-0.011~0.019	0.595
The overall regression F=37.648, P=0.000, the regression equation is significant.			

Note: *$P<0.05$, indicating that the regression coefficient of this variable is significant, **$P<0.01$, indicating that the regression coefficient of this variable is highly significant.

Supplementary Table 3. Multiple linear regression analysis of each right renal structural parameter and basic indices

**Renal length**	**β(SE)**	**95% CI**	**P-value**
Intercept	30.619(3.317)	24.100~37.138	0.000**
gender	1.300(0.672)	-0.020~2.621	0.054
age(year)	0.283(0.270)	-0.247~0.812	0.295
height(cm)	0.360(0.043)	0.275~0.445	0.000**
weight(kg)	0.112(0.043)	0.027~0.197	0.010**

The overall regression F=403.184, P=0.000, the regression equation is significant.

**Renal width**	**β(SE)**	**95% CI**	**P-value**
Intercept	21.460(1.561)	18.391~24.529	0.000**
gender	-1.187(0.316)	-1.808~-0.565	0.000**
age(year)	0.215(0.127)	-0.034~0.465	0.09
height(cm)	0.081(0.020)	0.041~0.122	0.000**
weight(kg)	0.150(0.020)	0.110~0.190	0.000**

The overall regression F=302.755, P=0.000, the regression equation is significant.

**Renal thickness**	**β(SE)**	**95% CI**	**P-value**
Intercept	25.817(2.009)	21.868~29.766	0.000**
gender	-1.011(0.407)	-1.811~-0.211	0.013*
age(year)	0.151(0.163)	-0.170~0.471	0.357
height(cm)	0.137(0.026)	0.085~0.188	0.000**
weight(kg)	0.105(0.026)	0.054~0.157	0.000**

The overall regression F=230.216, P=0.000, the regression equation is significant.

renal volume	β(SE)	95% CI	P-value
Intercept	-9.897(7.747)	-25.143~5.349	0.202
gender	-1.207(1.589)	-4.333~1.919	0.448
age(year)	1.229(0.607)	0.034~2.424	0.0445*
height(cm)	0.464(0.100)	0.267~0.661	0.000**
weight(kg)	0.875(0.095)	0.688~1.062	0.000**

The overall regression F=226.585, P=0.000, the regression equation is significant. *P<0.05, indicating that the regression coefficient of this variable is significant, **P<0.01, indicating that the regression coefficient of this variable is highly significant.

renal cortical thickness	β(SE)	95% CI	P-value
Intercept	1.639(0.436)	0.781~2.496	0.000**
gender	0.084(0.088)	-0.090~0.257	0.345
age(year)	0.010(0.035)	-0.060~0.079	0.784
height(cm)	0.023(0.006)	0.012~0.034	0.000**
weight(kg)	0.020(0.006)	0.009~0.031	0.000**

The overall regression F=132.986, P=0.000, the regression equation is significant. **P<0.01, indicating that the regression coefficient of this variable is highly significant.

Renal artery diameter	β(SE)	95% CI	P-value
Intercept	2.027(0.554)	0.937~3.116	0.000**
gender	0.029(0.112)	-0.191~0.250	0.795
age(year)	0.099(0.045)	0.011~0.188	0.028*
height(cm)	0.008(0.007)	-0.006~0.022	0.272

weight(kg)	-0.001(0.007)	-0.015~0.014	0.931
The overall regression F=34.189, P=0.000, the regression equation is significant.			

Note: *P<0.05, indicating that the regression coefficient of this variable is significant, **P<0.01, indicating that the regression coefficient of this variable is highly significant.
