# Supplementary material for: The normal reference values and estimation formulae of renal structural parameters in Chinese children based on large-sample CT data
Source: Front Pediatr. 2023 Jul 17;11:1174310. doi: 10.3389/fped.2023.1174310 (PMC10388191; doi:10.3389/fped.2023.1174310)
Supplement: Supplementary file 2 [file Image1.pdf]

Supplementary Material

The normal reference values and estimation formulae of renal structural parameters in Chinese children based on large-sample CT data

Yong Qin, En Liu, Xiaoying Ni, Zhongxin Huang, Lu Tian, Xiaoya He, Jinhua Cai*, Qiu Li*.

1* Correspondence: Liqiu809@hospital.cqmu.edu.cn

2* Correspondence: cai_jinhua@126.com

1 Supplementary Images

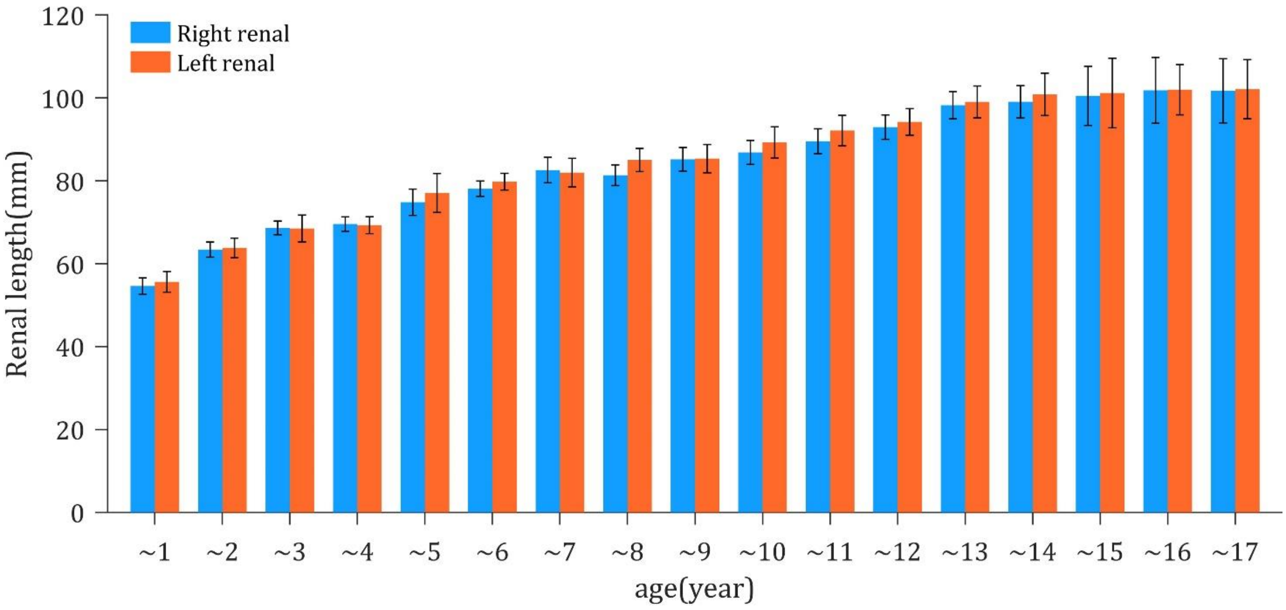

Supplementary Image 1.1 Trends of RL of the right and left kidney with age.

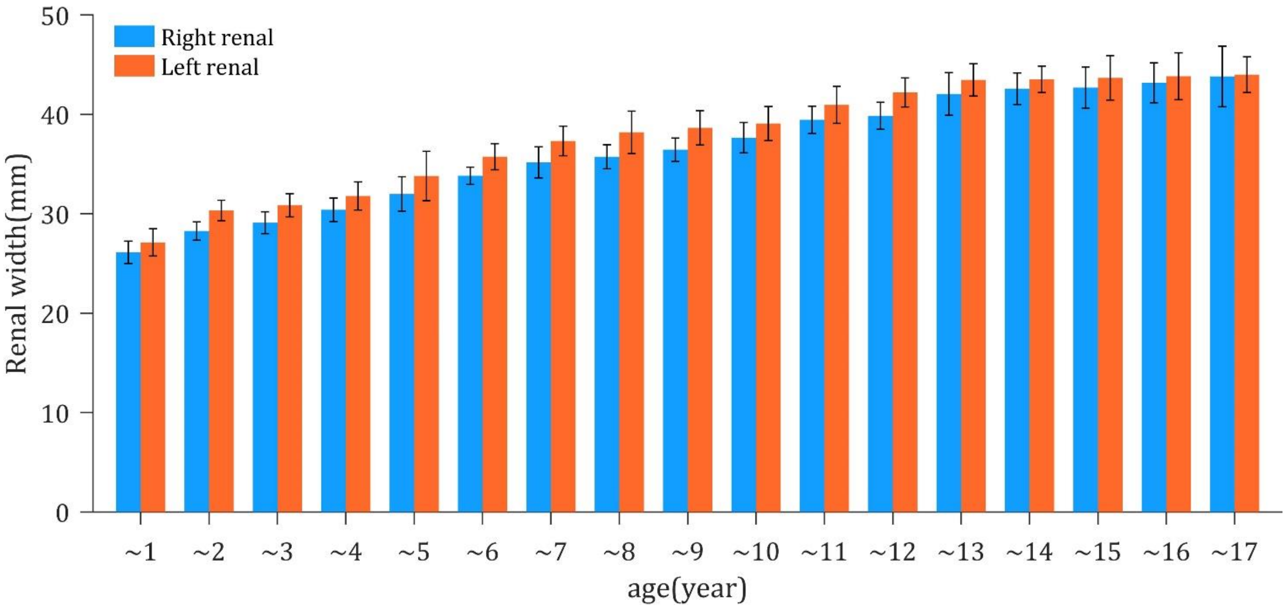

Supplementary Image 1.2 Trends of RW of the right and left kidney with age.

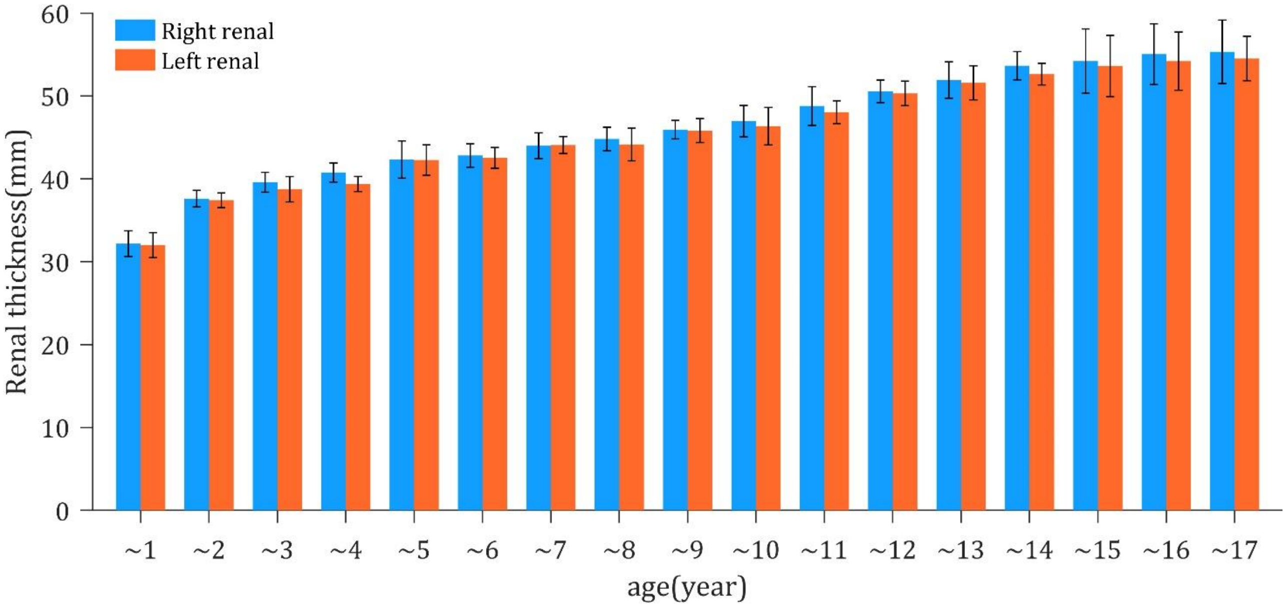

Supplementary Image 1.3 Trends of RT of the right and left kidney with age.

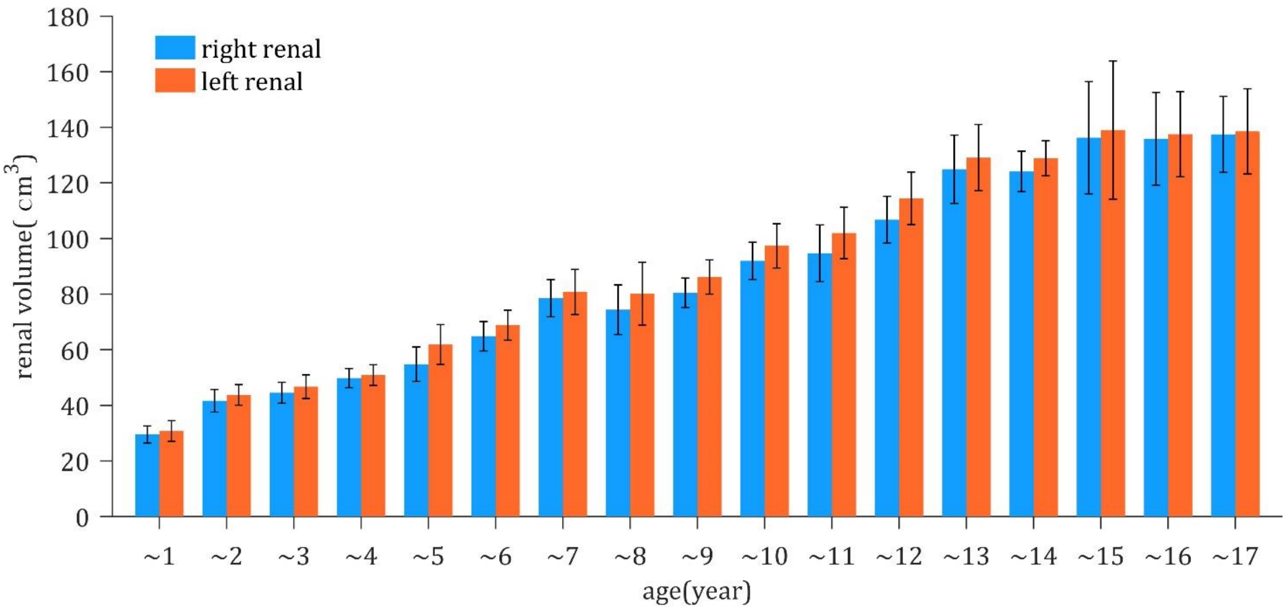

Supplementary Image 1.4 Trends of RV of the right and left kidney with age.

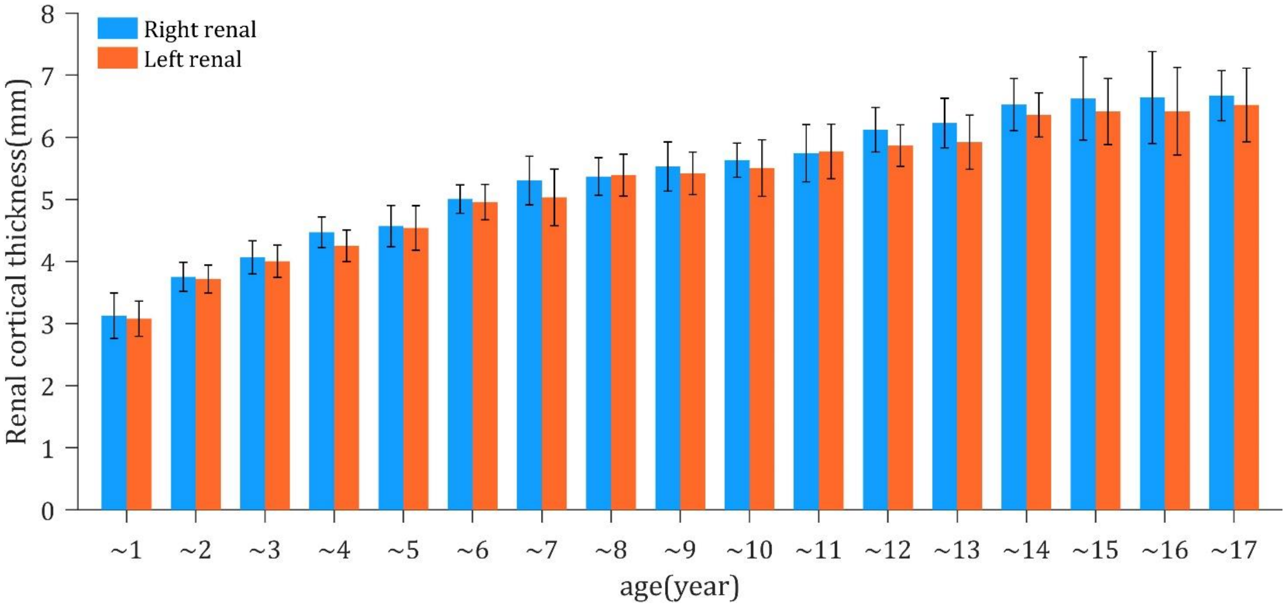

Supplementary Image 1.5 Trends of RCT of the right and left kidney with age.

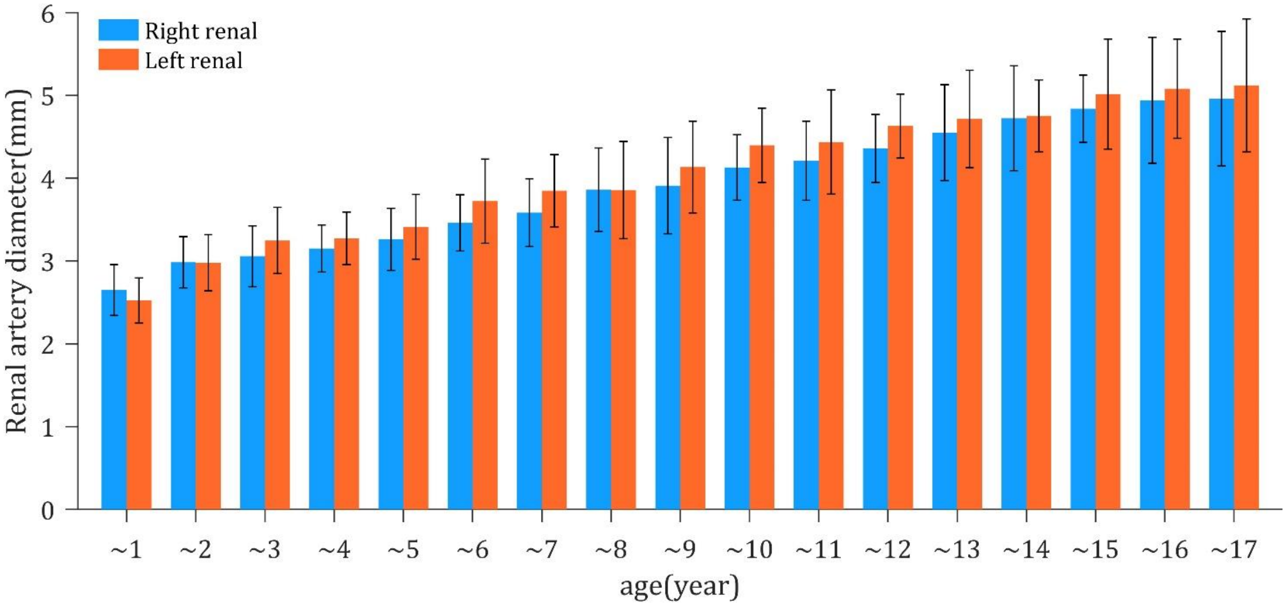

Supplementary Image 1.6 Trends of RAD of the right and left kidney with age.

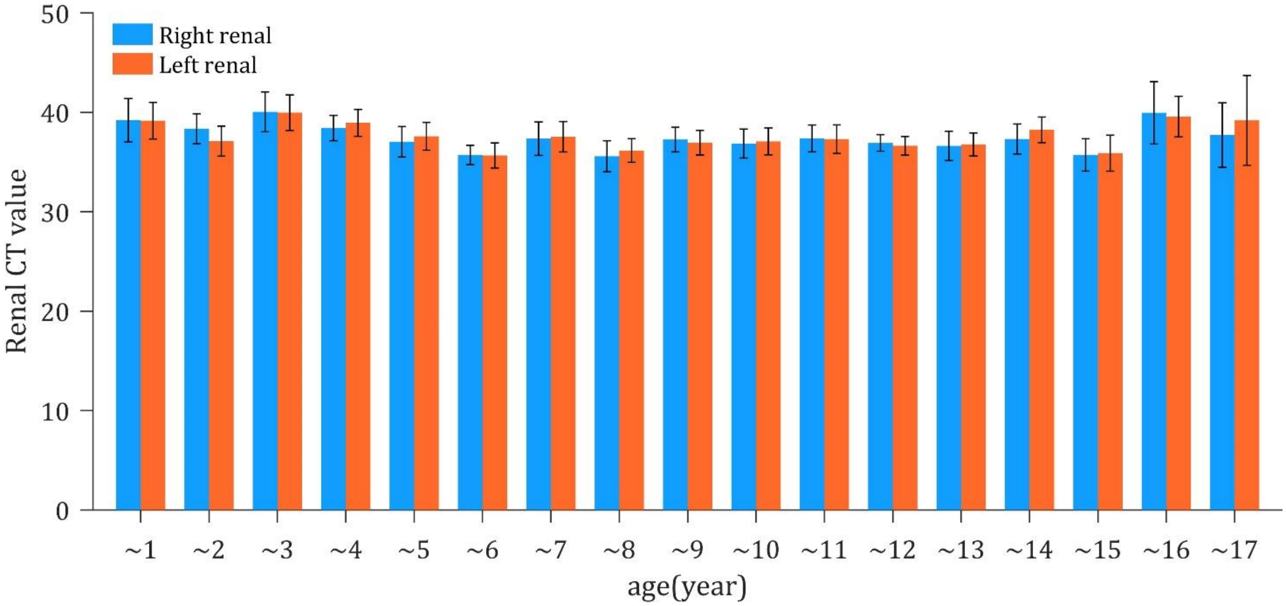

Supplementary Image 1.7 Trends of CT values of the right and left kidney with age.
